# Supplementary material for: Slip bursts during coalescence of slow slip events in Cascadia
Source: Nat Commun. 2020 May 1;11:2159. doi: 10.1038/s41467-020-15494-4 (PMC7195424; doi:10.1038/s41467-020-15494-4)
Supplement: Supplementary file 1 — Supplementary Information [file 41467_2020_15494_MOESM1_ESM.pdf]

Supplementary Information for:

Slip bursts during coalescence of slow slip events in Cascadia

by Bletery and Nocquet

Supplementary Notes

Supplementary Note 1: Data fit

The prediction of our model is in good agreement with the 124 observed GPS time series (Supplementary Figures 7-17). We note that the formal errors on the daily coordinates provided with the GAGE combination center (*39*) (blue and black error bars in Supplementary Figures 7-17) exceeds significantly the observed daily scatter. In order to use more realistic formal errors in our resolution test, we selected 12 stations which show no obvious signal during the studied period (BCOV, HOLB, LINH, P410, P413, P416, P420, P421, P431, P432, P434, P705) and calculated the difference of position from one day to the next. We averaged the obtained values across all 12 stations (weighted by their respective number of points) and found standard deviations of 0.81 mm for the east component and 0.94 mm for the north component. Most studies conclude that for periods shorter than a few months as it is the case here, a white noise model correctly explains the GPS residual time series (*40*). Therefore, our procedure allows to generate time series consistent with the actual precision of the GAGE time series over the studied area.

Supplementary Note 2: Resolution tests

To evaluate the resolution power of our approach we designed a simple resolution test. We built a synthetic model composed of 3 patches roughly corresponding to the 3 ruptures fronts imaged in the inversion before their merging. Each patch is composed of 13 subfaults and starts slipping at a different time (Supplementary Figures 19 (left), 20, 21 (solid lines)). Slip is maximal at the center of each patch, 50% of the maximum value on the 3 adjacent subfaults and 20% on the next row. Slip increases continuously following an arc-tangent function from 0 to a maximum value (Supplementary Figure 21 (solid lines)) which is set to 2 mm to match the overall moment obtained in our inversion of the actual data. We computed the prediction

of the GPS time series from this synthetic model and added Gaussian noise centered in 0 with standard deviations of 0.81 mm in the east component and 0.94 mm in the north component (as evaluated in Supplementary Note 1). We inverted the obtained synthetic data following the same procedure as in the original inversion. We performed a set of inversions using different values of σ and found an optimal value of $\sigma = 5 \text{ mm.day}^{-1/2}$ (Supplementary Figure 18). The final cumulative slip distribution is well recovered (Supplementary Figure 19). The time evolution is also well recovered for each patch : the inverted slip initiates on each patch at the time of the target synthetic model (dashed versus solid lines in Supplementary Figures 20-21). We observe no noticeable slip recovered outside of the 3 synthetic patches (black dashed line in Supplementary Figures 20-21). We note a general under-prediction of the recovered slip (Supplementary Figure 22), particularly important on the northern patch (Supplementary Figure 20), suggesting that the amount of slip we imaged in the original inversion might be under-estimated too, in particular in this section. We also see that the recovered source time functions is slightly more irregular than the target (Supplementary Figure 22) but the amplitude of the oscillations is orders of magnitudes smaller than what we see in the main inversion, indicating that the shape of Fig. 3 is constrained by the data.

Supplementary Figures

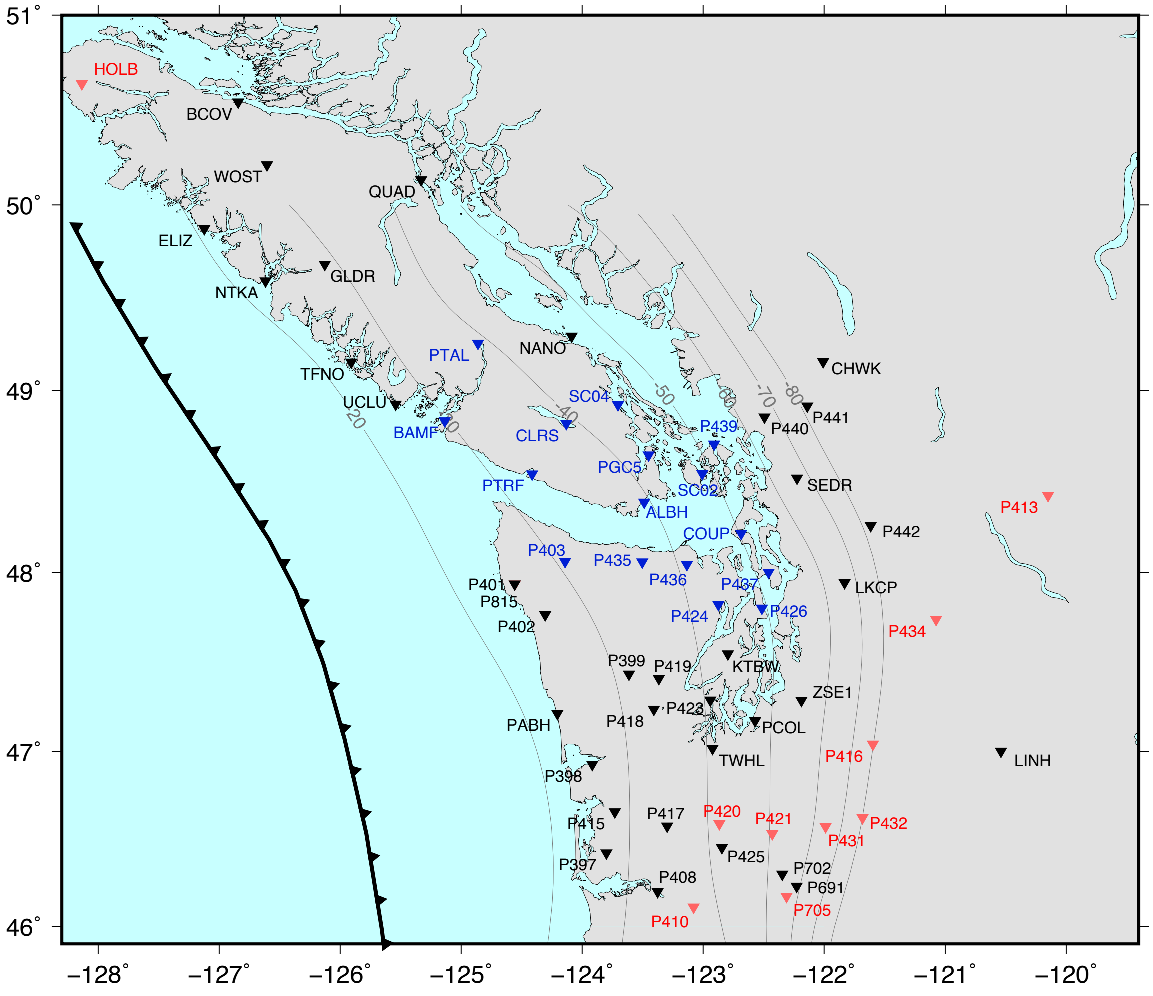

Supplementary Figure 1: Location of GPS stations used in the inversion. Stations represented in blue and red are used in the calculation of the stacked daily slip change inside and away from the merging area, respectively (Fig. 3.b).

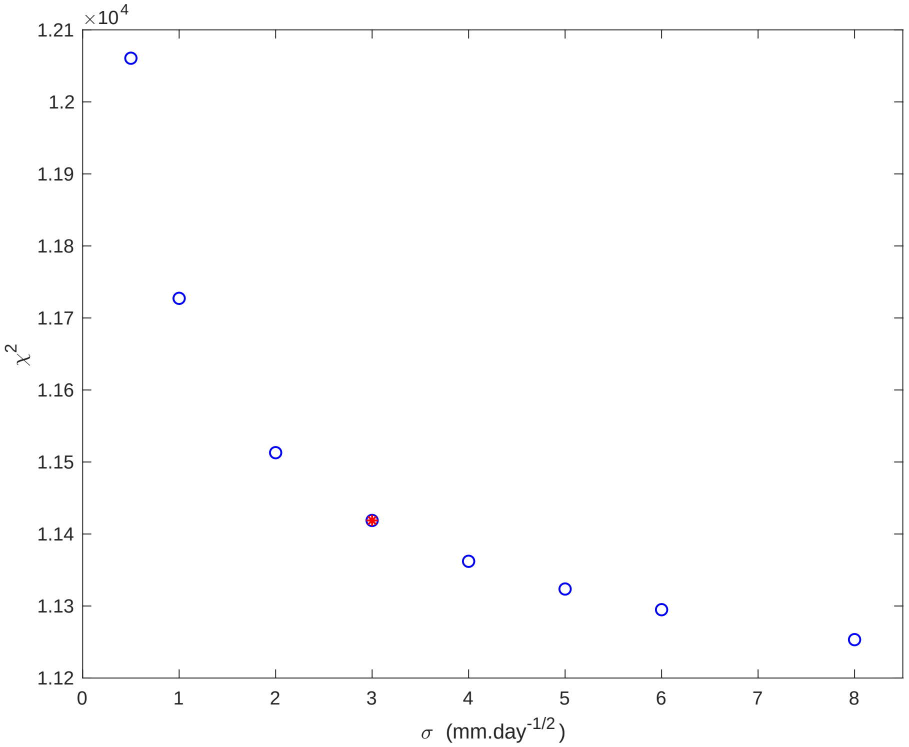

Supplementary Figure 2: Misfit of the inversion as a function of the regularization parameter σ, commonly known as L-curve. The red star indicates $\sigma = 3\ \mathrm{mm.day}^{-1/2}$ (value used in the inversion).

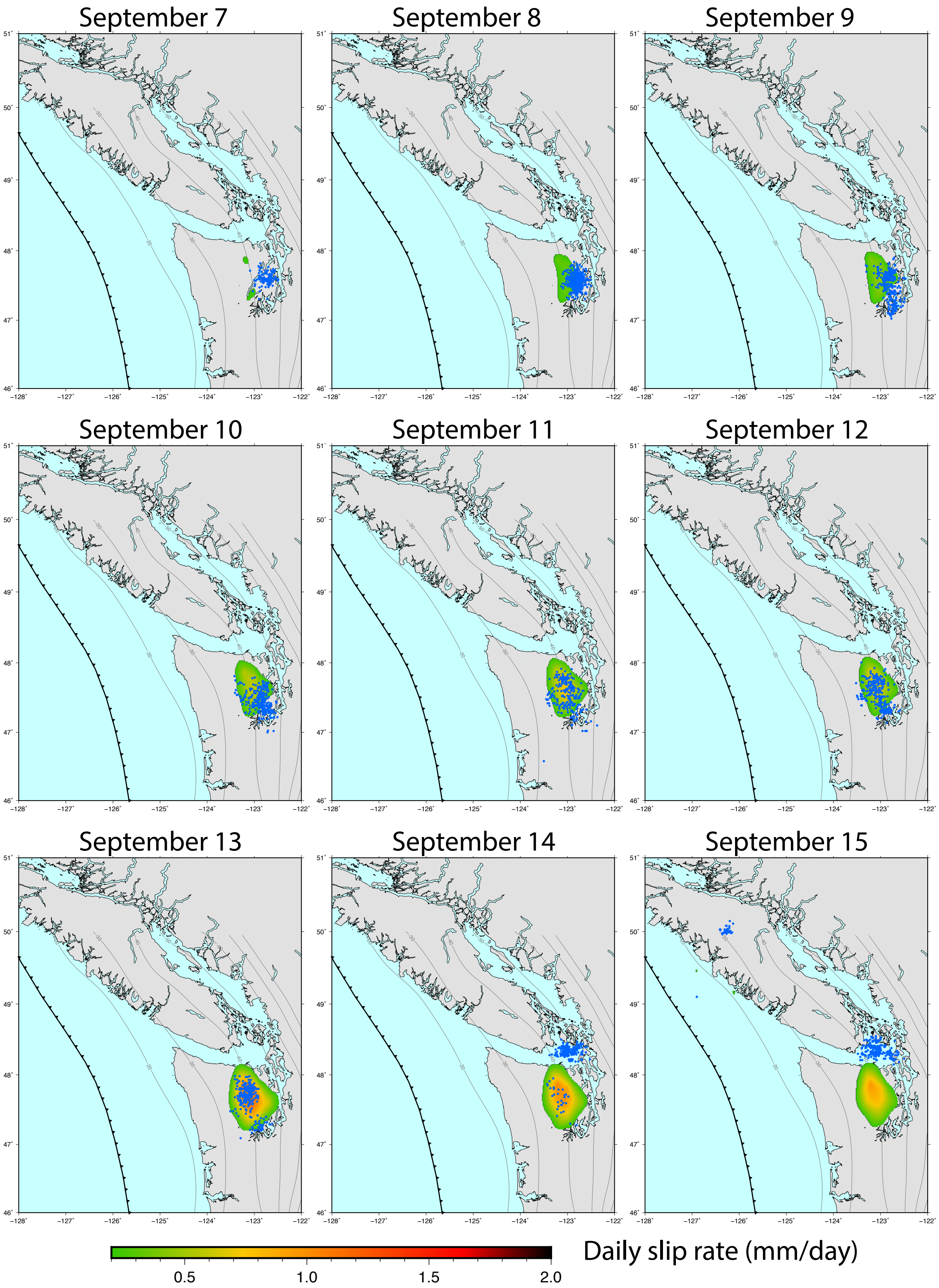

Supplementary Figure 3: Inverted daily slip along the megathrust from September 7 to September 15 2013. Blue dots show daily recorded tremors.

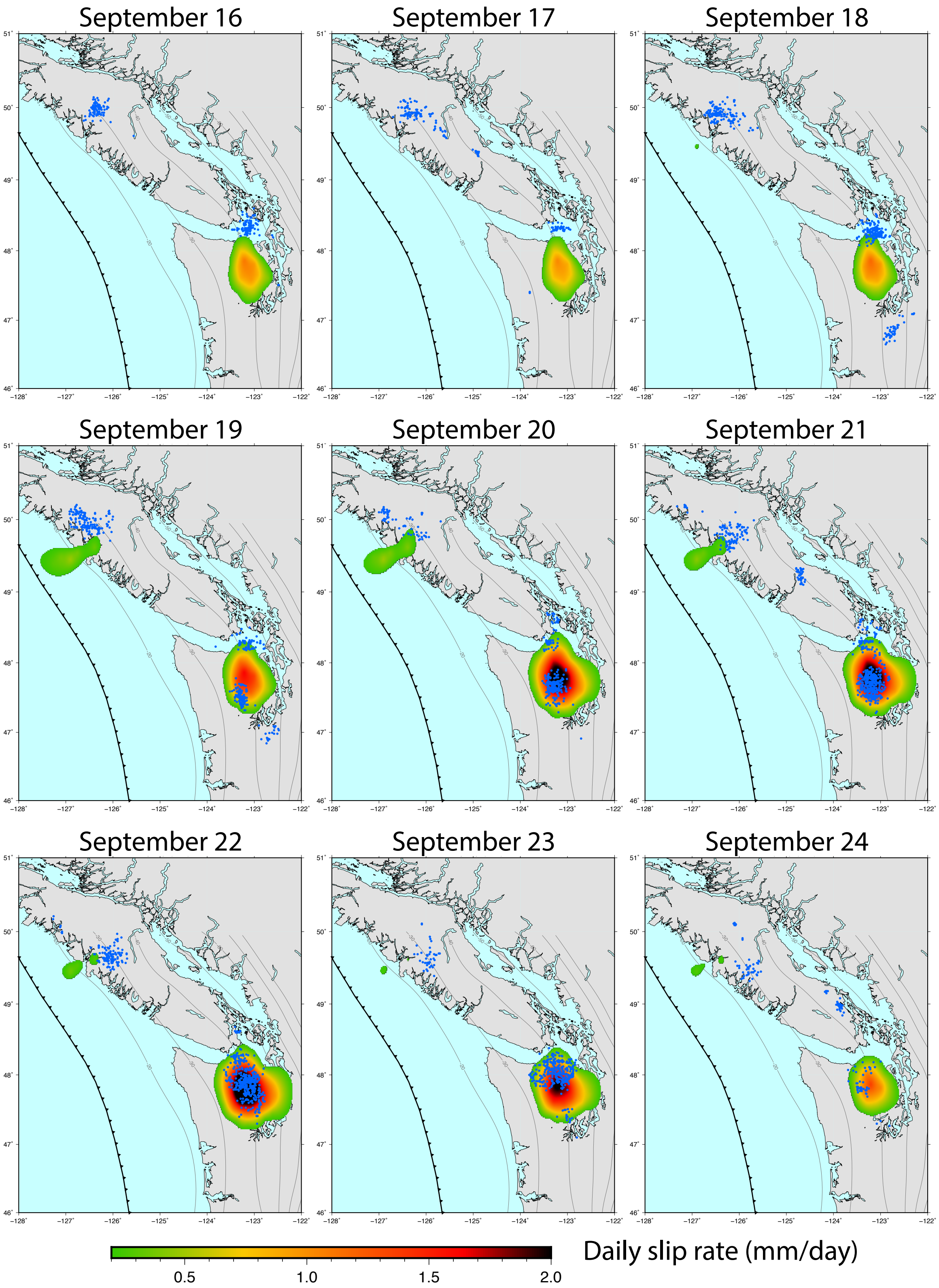

Supplementary Figure 4: Inverted daily slip along the megathrust from September 16 to September 24 2013. Blue dots show daily recorded tremors.

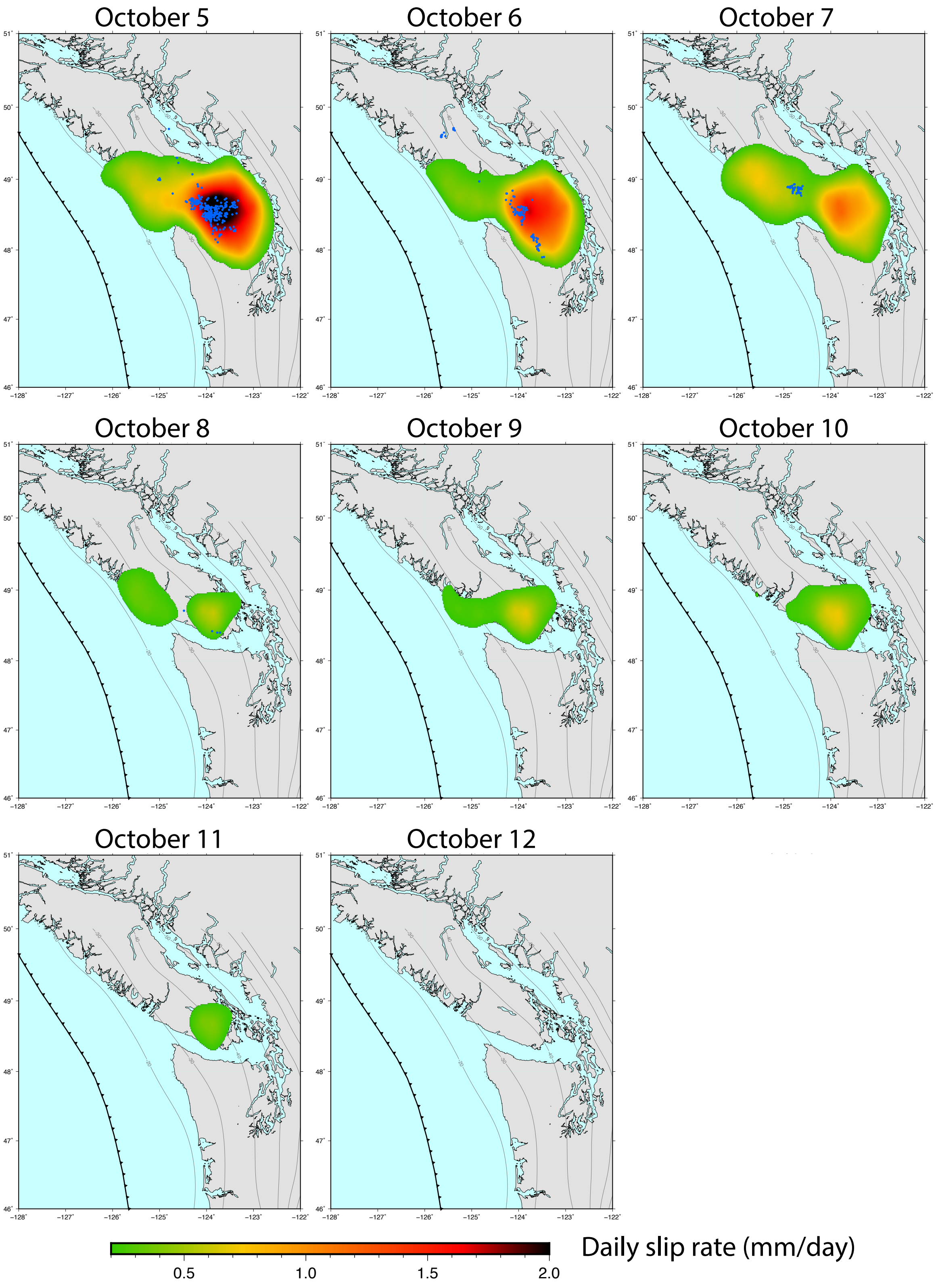

Supplementary Figure 5: Inverted daily slip along the megathrust from October 5 to October 12 2013. Blue dots show daily recorded tremors.

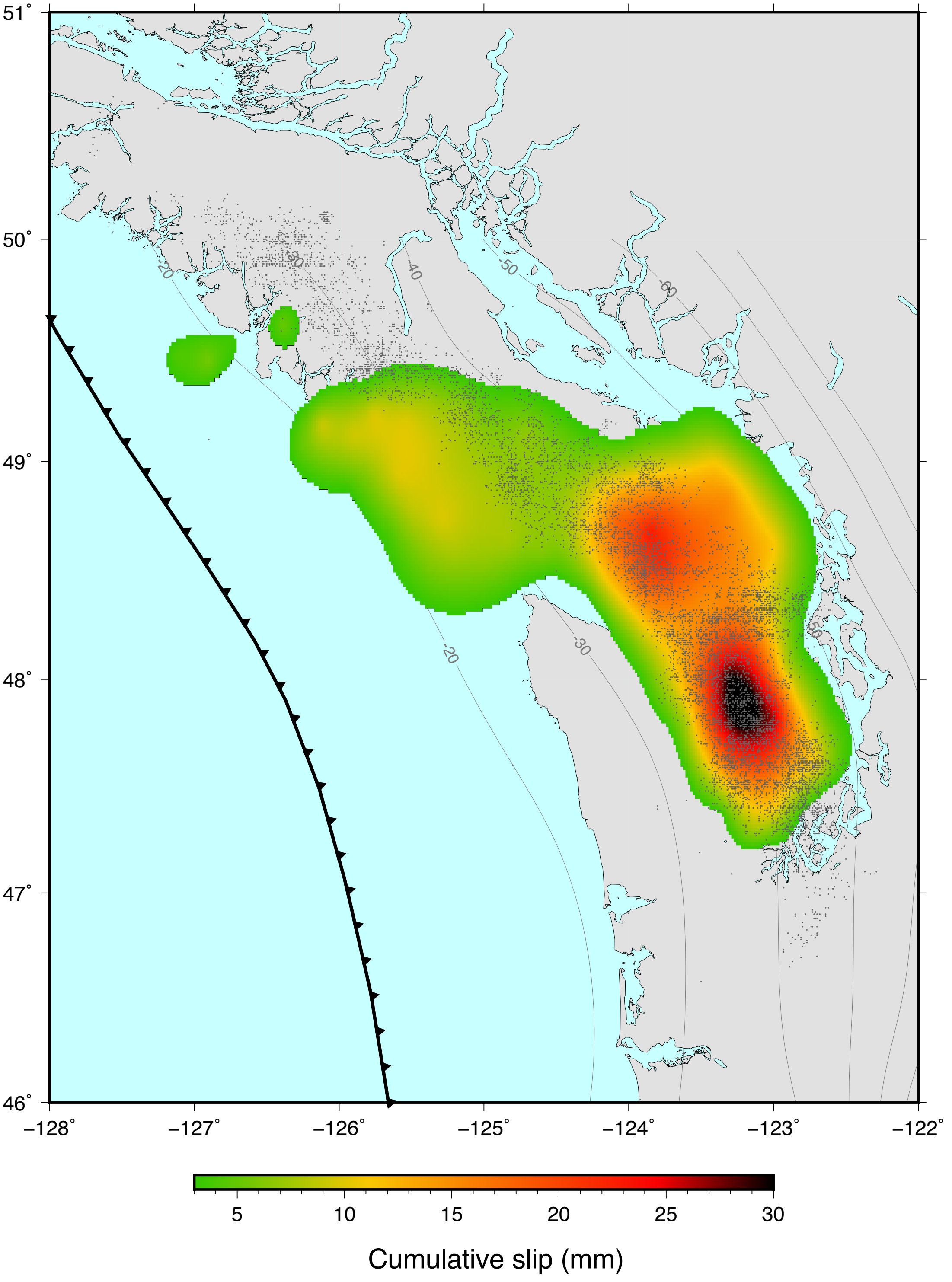

Supplementary Figure 6: Cumulative slip distribution inverted along the megathrust from August 27 to October 12 2013. Gray dots show daily recorded tremors.

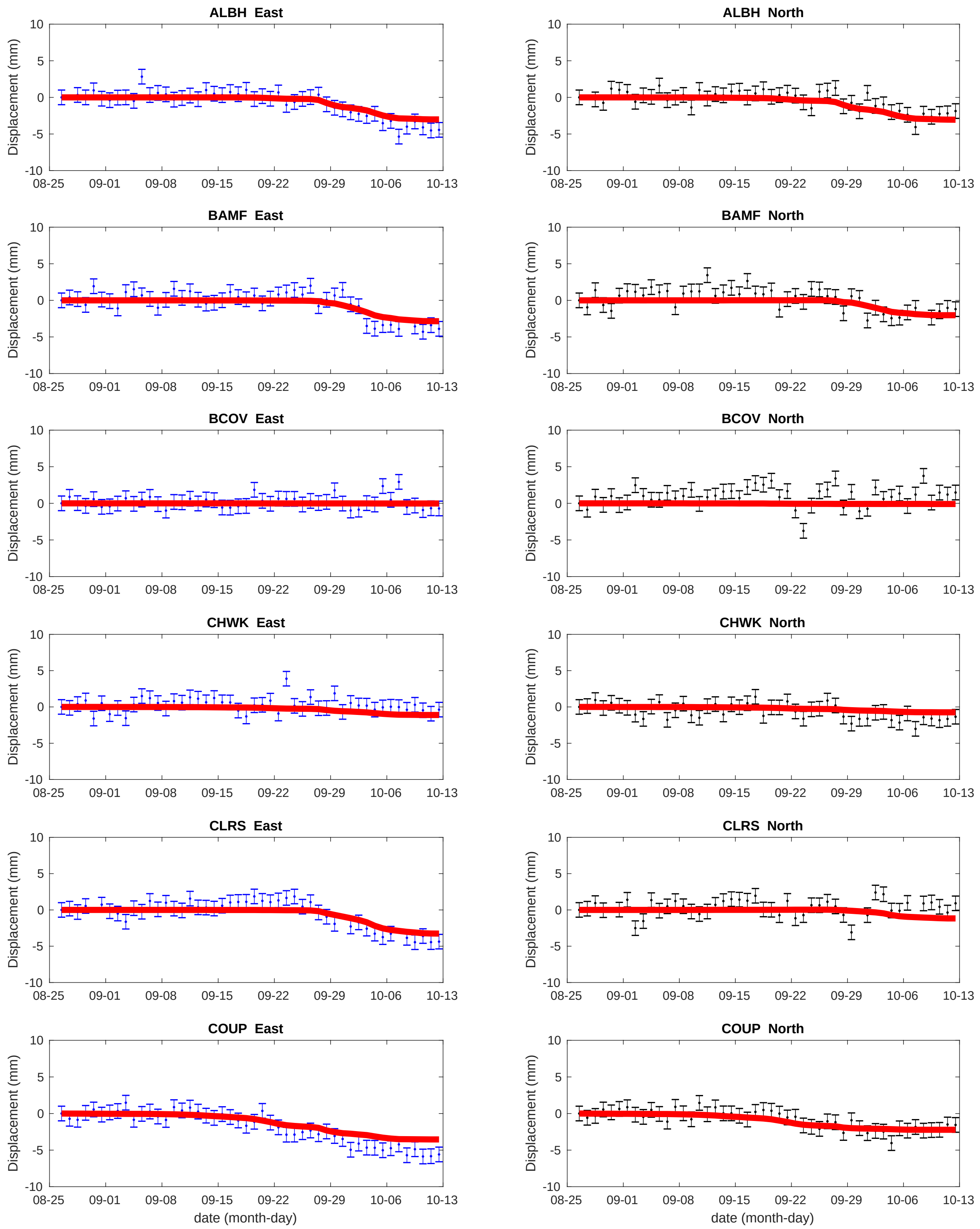

Supplementary Figure 7: Observed and predicted GPS time series. Blue shows measurements on the East component with their uncertainty (provided by UNAVCO). Black shows measurements on the North component. Red is the prediction of the inverted slip model. Stations ALBH, BAMF, BCOV, CHWK, CLRS, COUP.

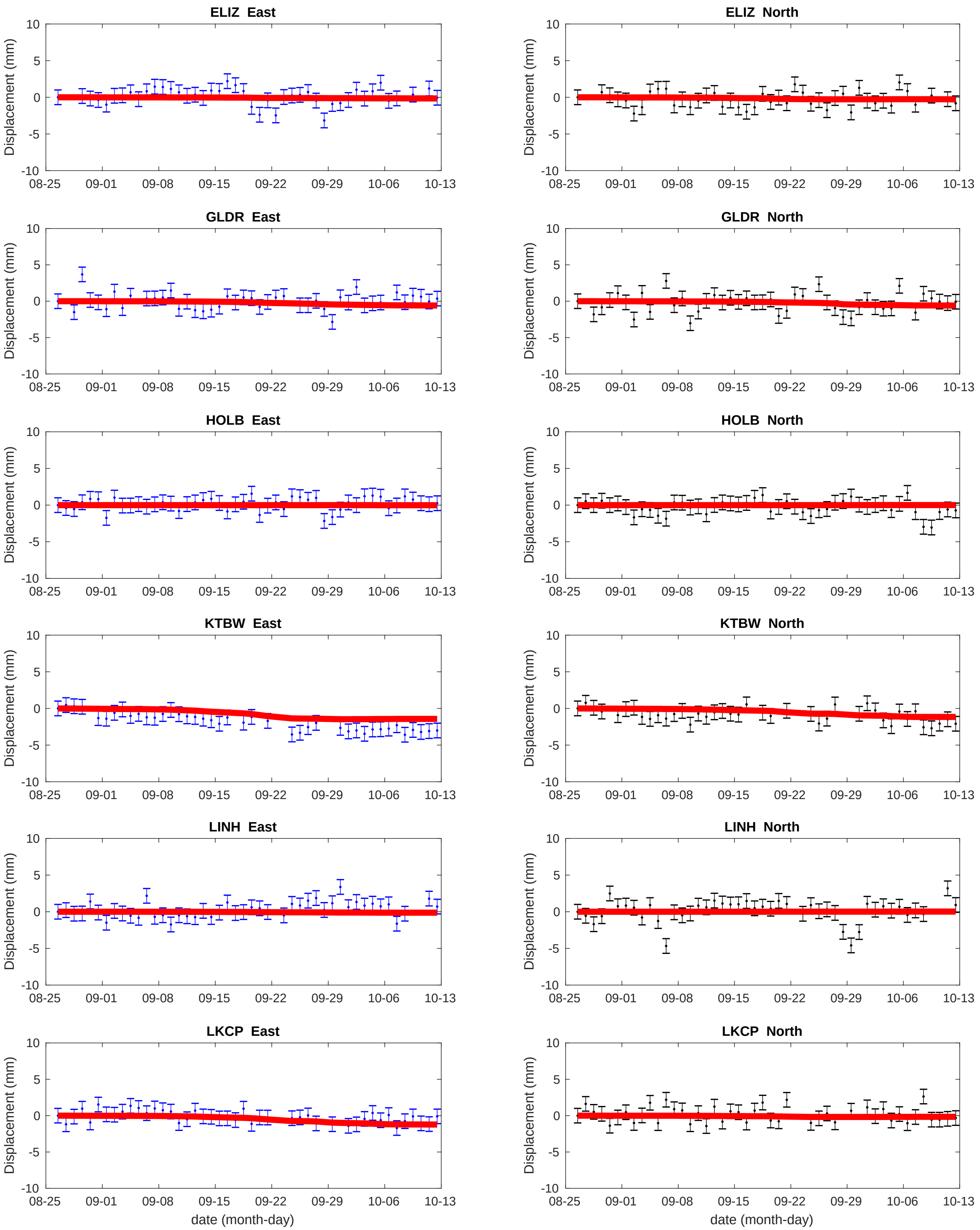

Supplementary Figure 8: Data fit. Stations ELIZ, GLDR, HOLB, KTBW, LINH, LKCP.

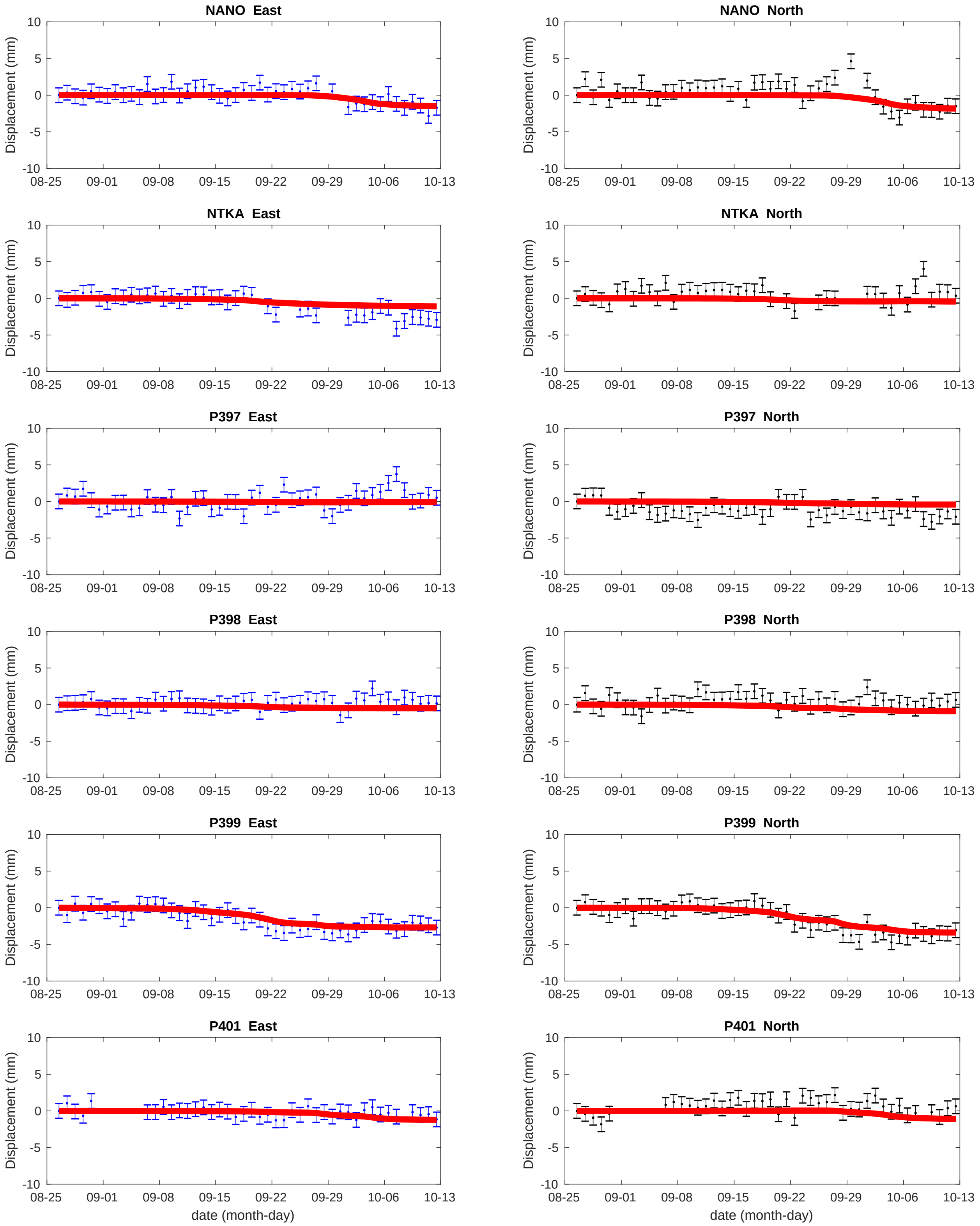

Supplementary Figure 9: Data fit. Stations NANO, NTKA, P397, P398, P399, P401.

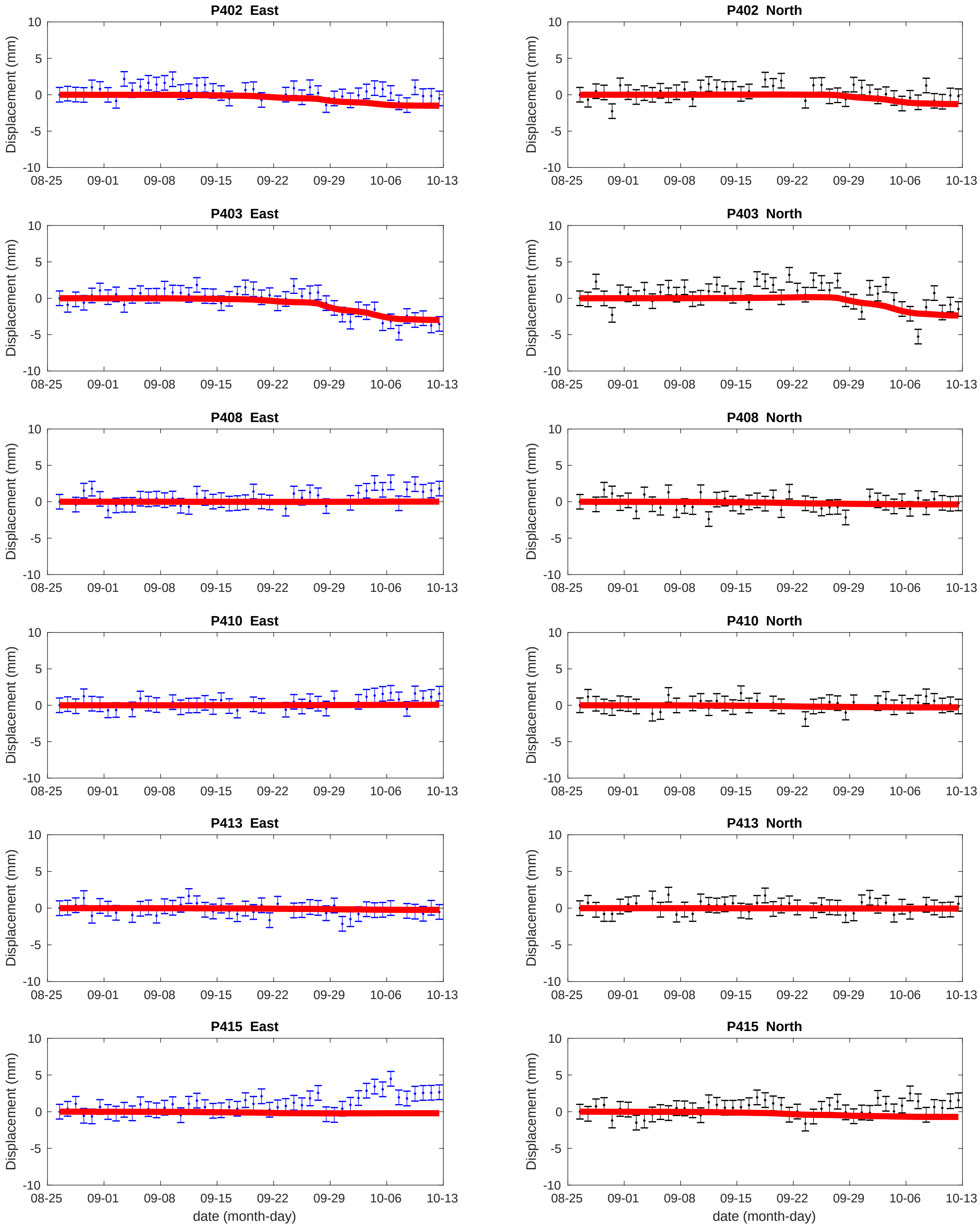

Supplementary Figure 10: Data fit. Stations P402, P403, P408, P410, P413, P415.

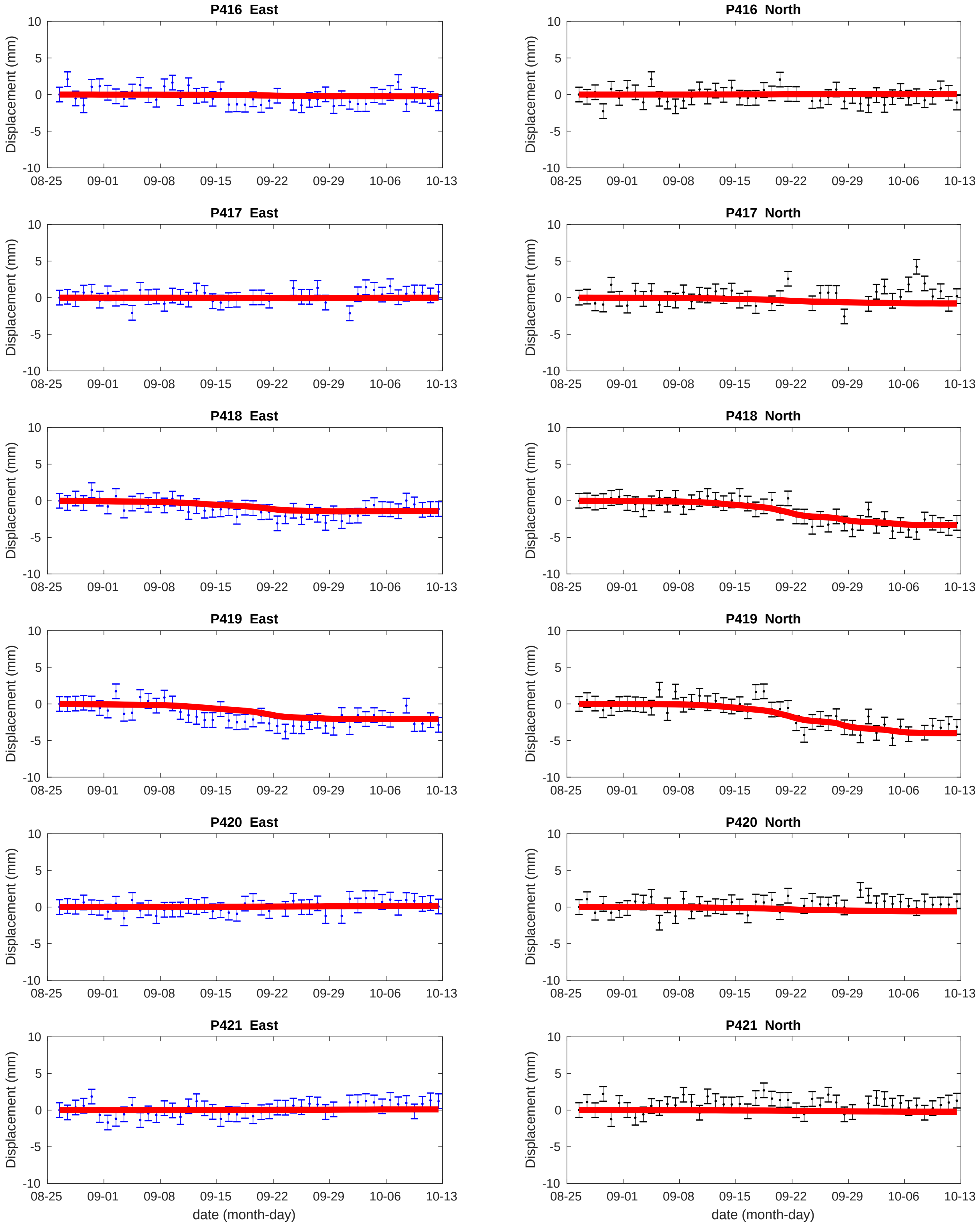

Supplementary Figure 11: Data fit. Stations P416, P417, P418, P419, P420, P421.

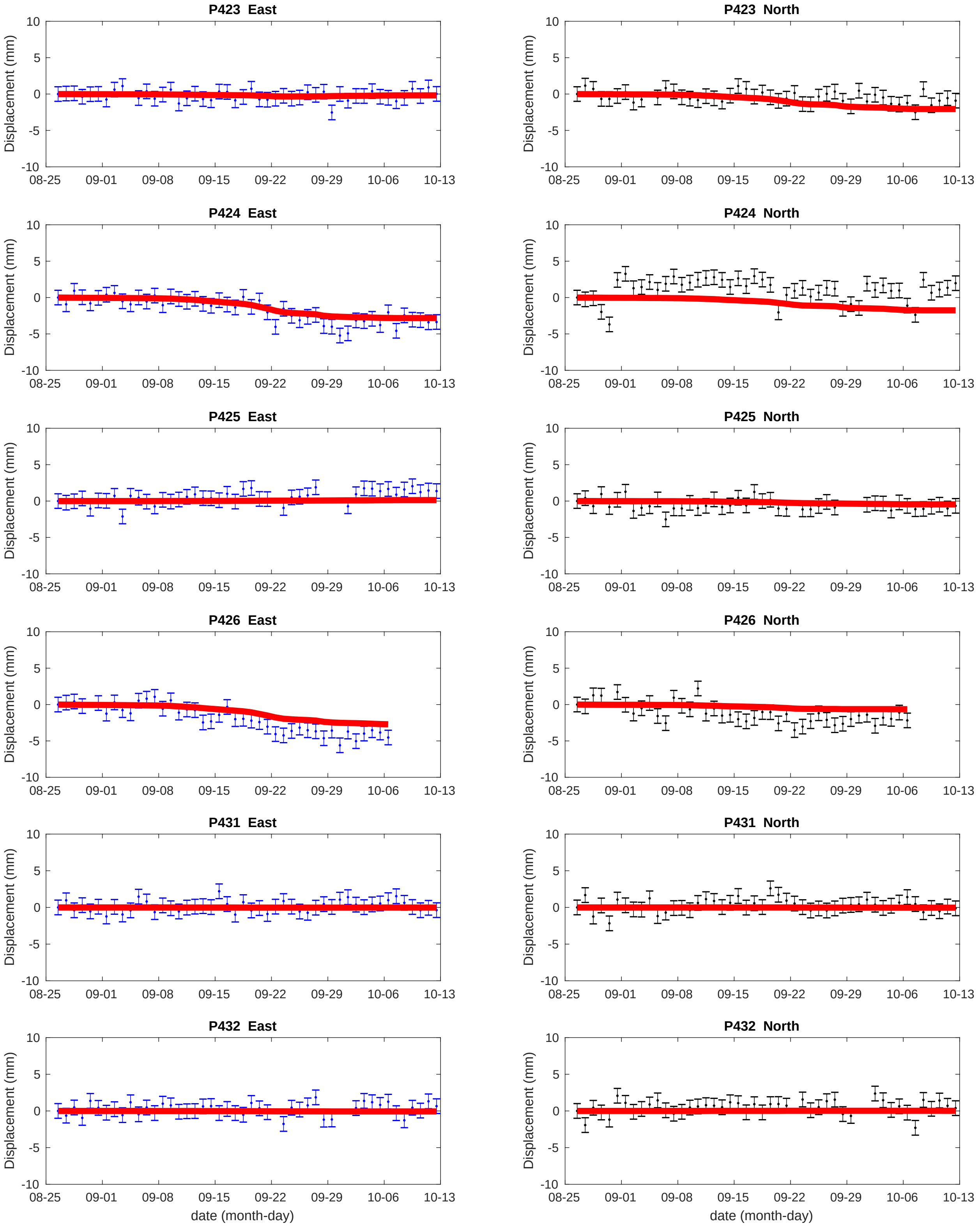

Supplementary Figure 12: Data fit. Stations P423, P424, P425, P426, P431, P432.

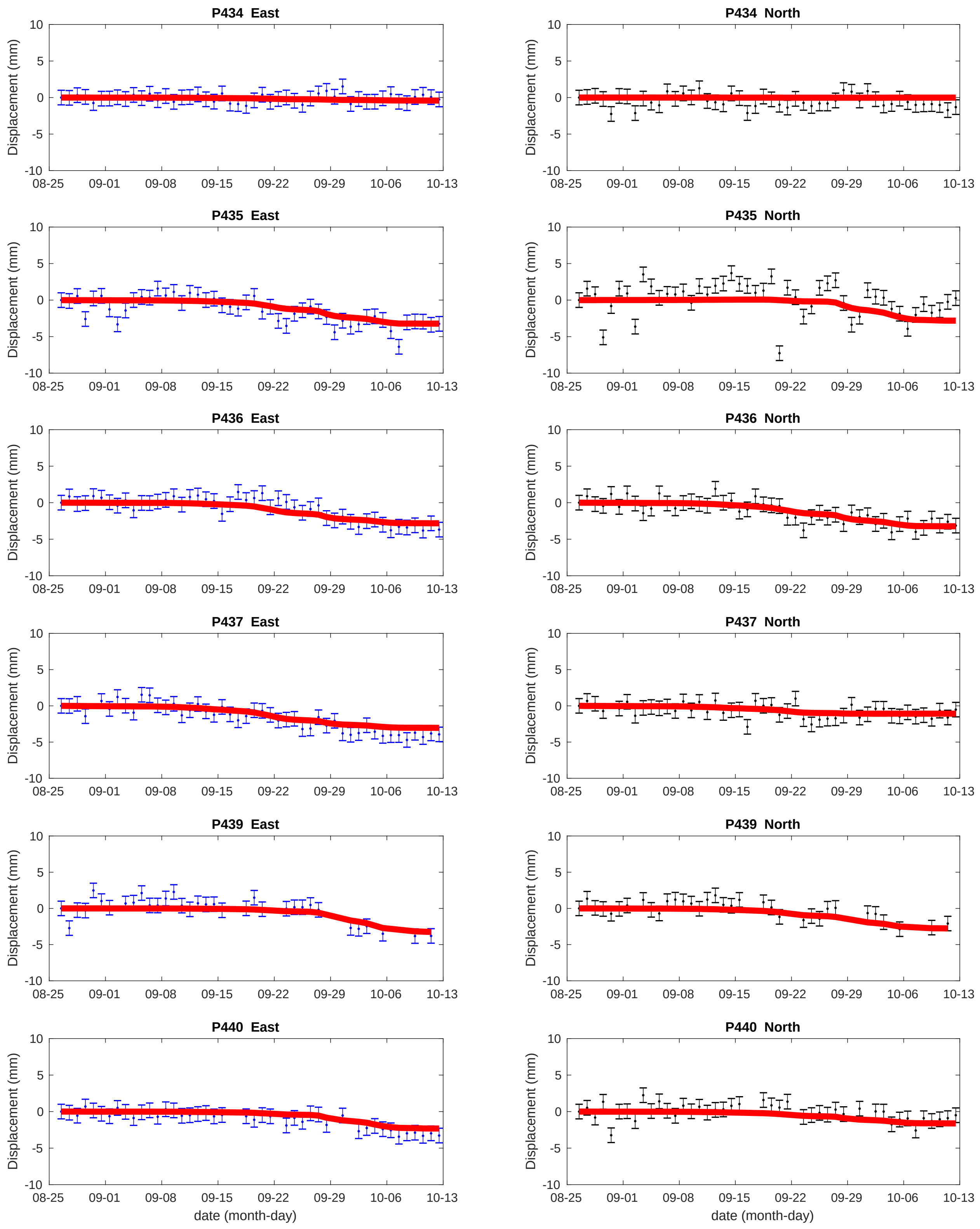

Supplementary Figure 13: Data fit. Stations P434, P435, P436, P437, P439, P440.

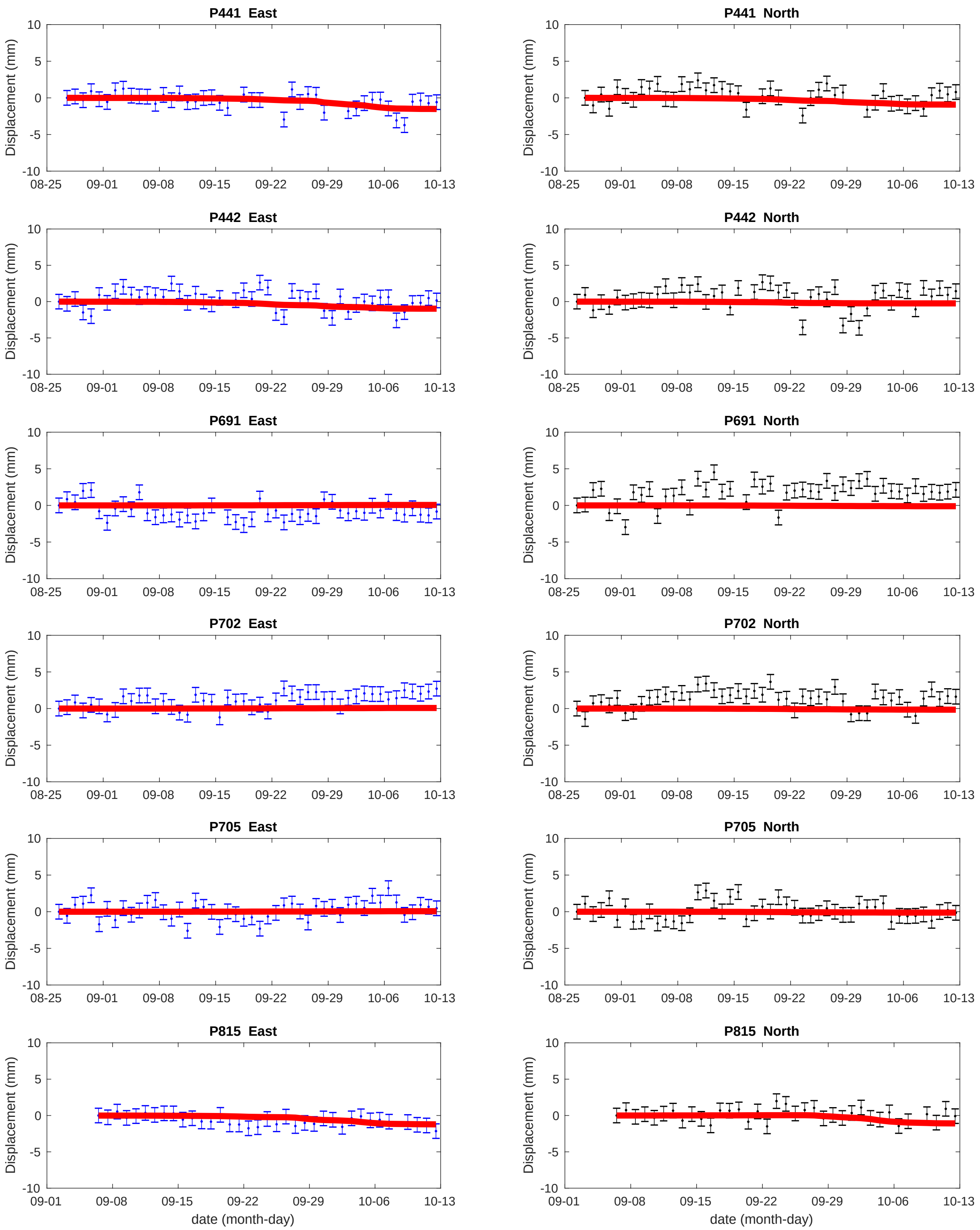

Supplementary Figure 14: Data fit. Stations P441, P442, P691, P702, P705, P815.

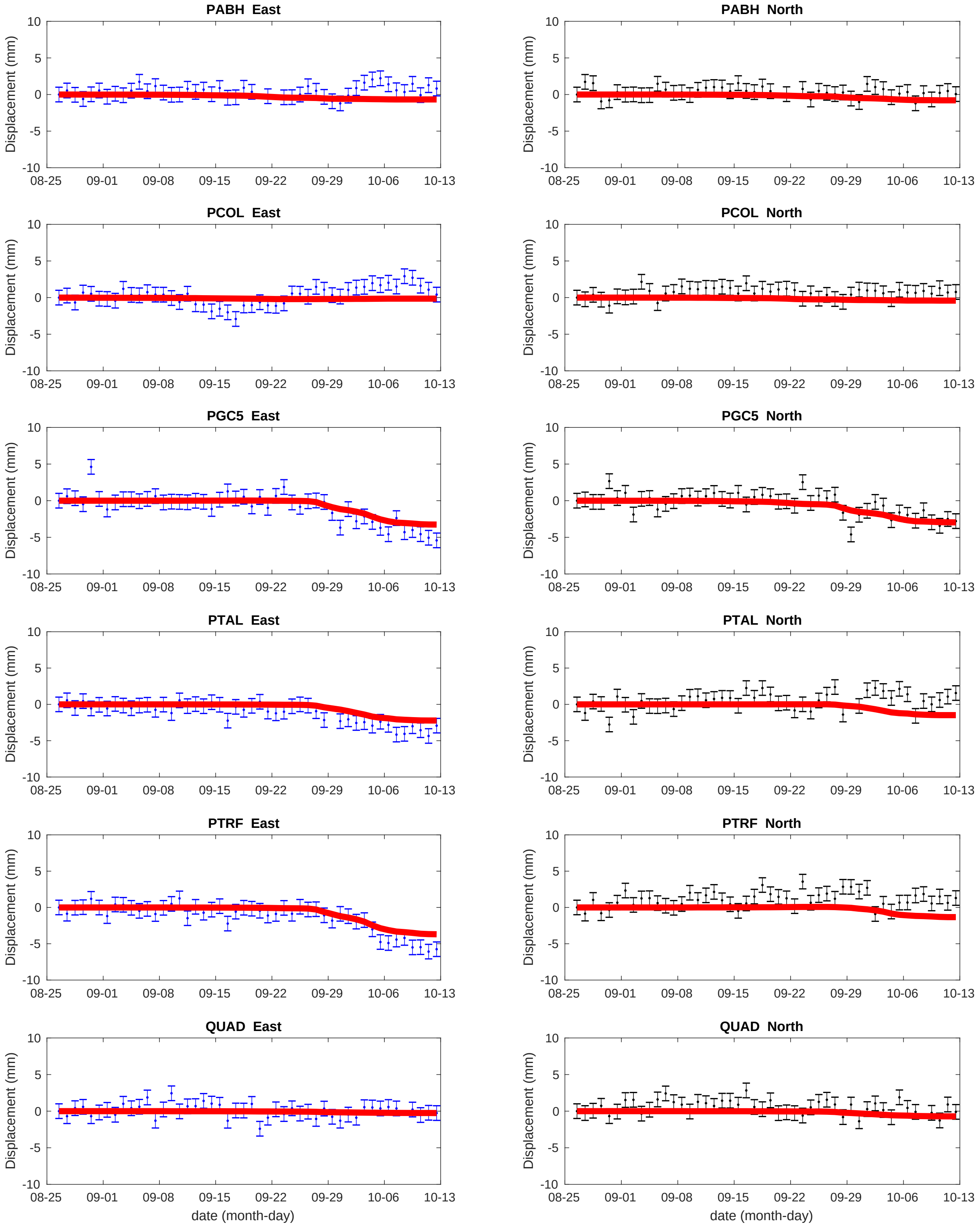

Supplementary Figure 15: Data fit. Stations PABH, PCOL, PGC5, PTAL, PTRF, QUAD.

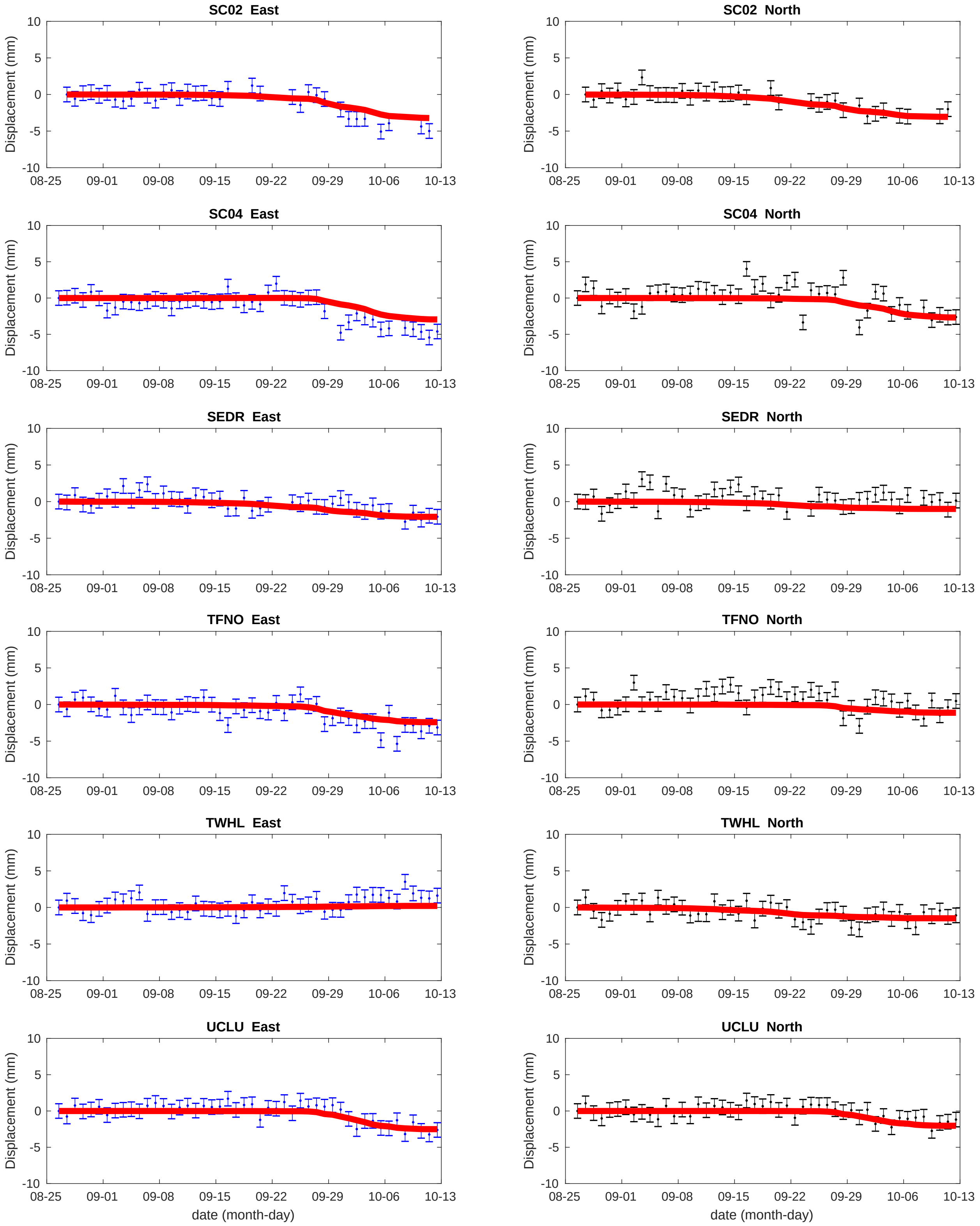

Supplementary Figure 16: Data fit. Stations SC02, SC04, SEDR, TFNO, TWHL, UCLU.

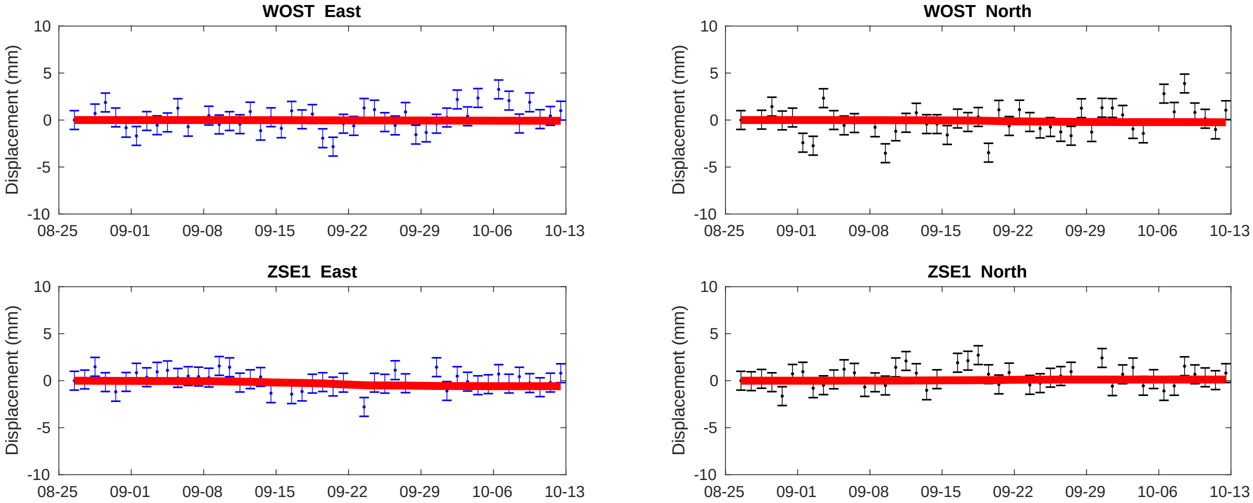

Supplementary Figure 17: Data fit. Stations WOST, ZSE1.

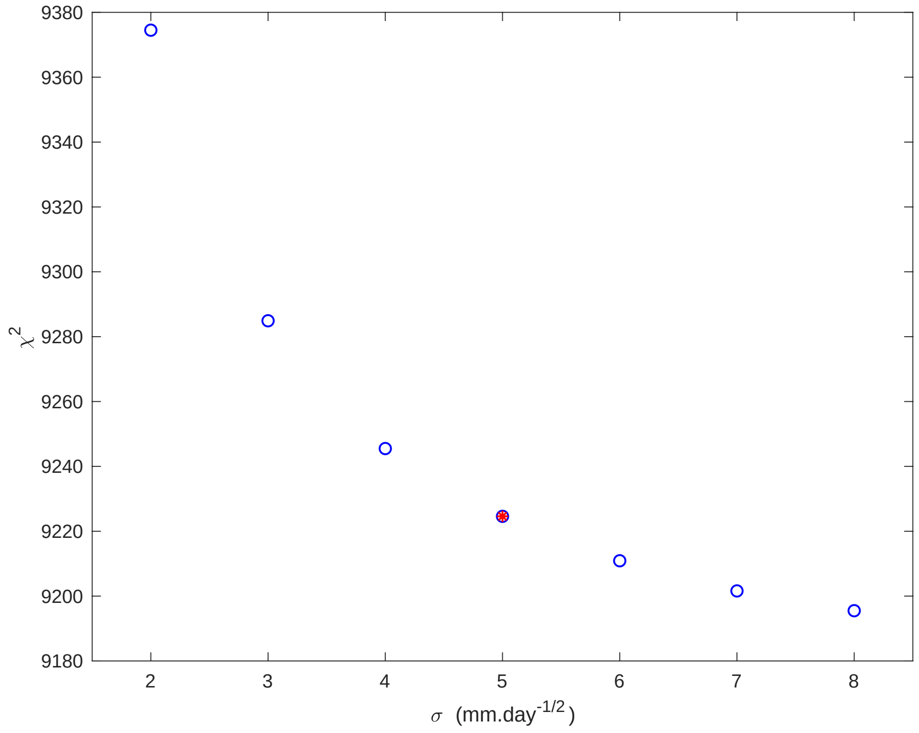

Supplementary Figure 18: L-curve for the resolution test. The red star indicates $\sigma = 5$ $\mathrm{mm.day}^{-1/2}$ (value used in the resolution test).

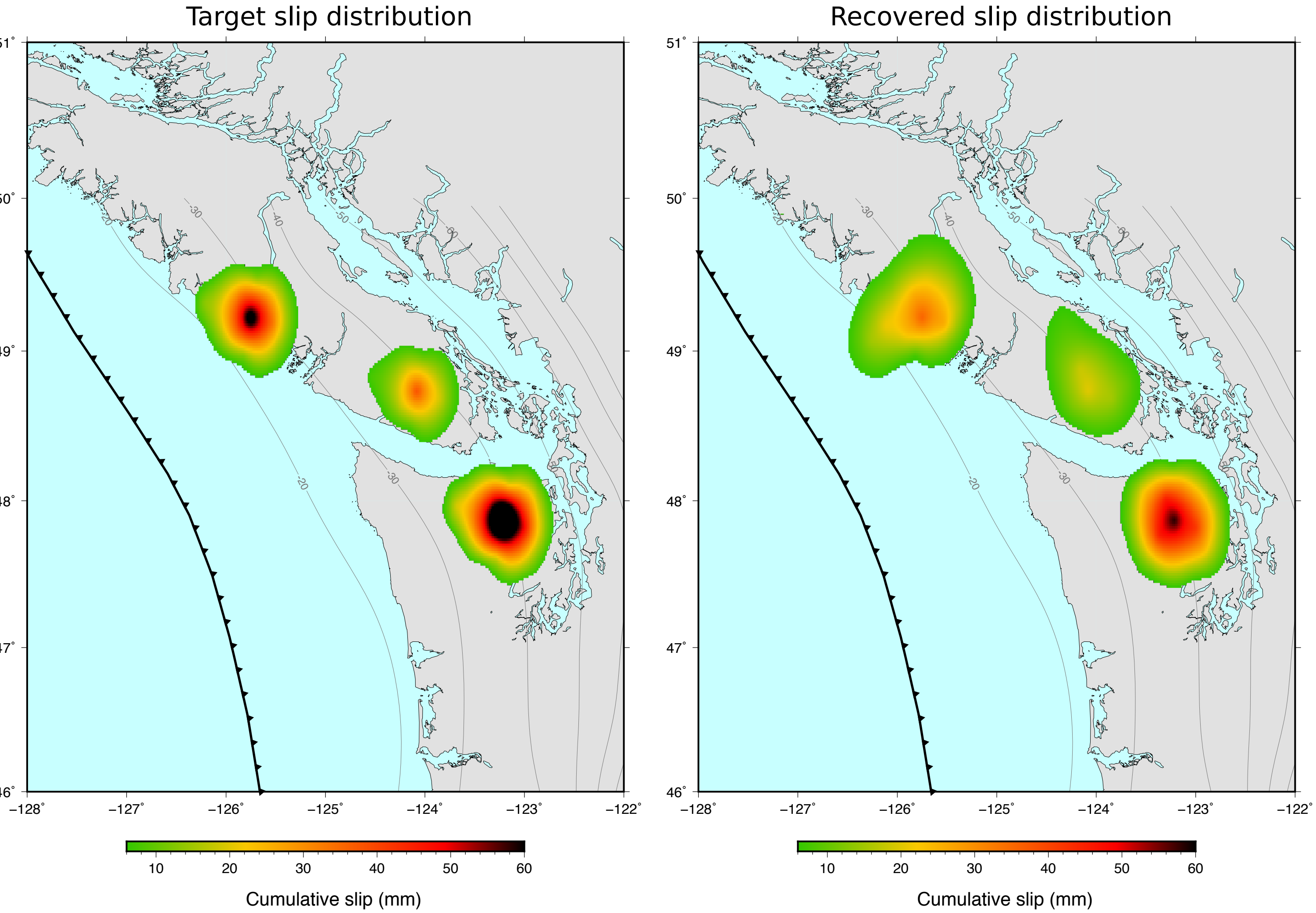

Supplementary Figure 19: Target (left) and recovered (right) final cumulative slip distribution for the resolution test.

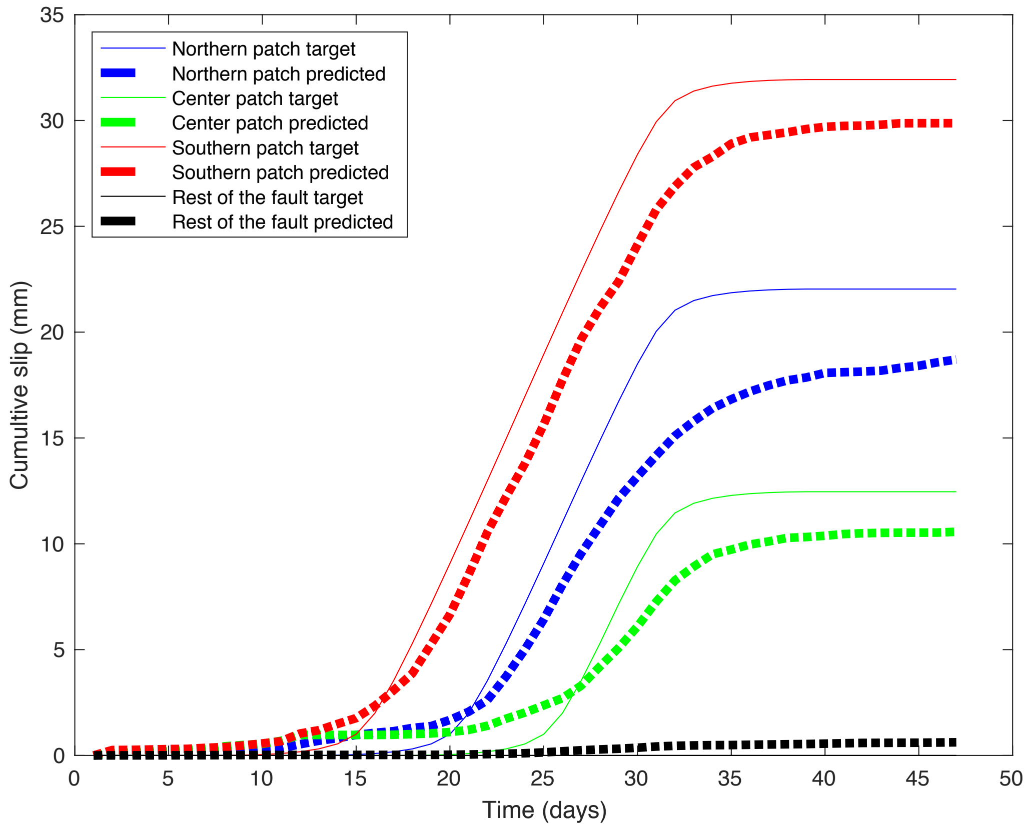

Supplementary Figure 20: Target (plain lines) and recovered (dashed lines) cumulative slip time series for the 3 patches in the resolution test and the rest of the fault.

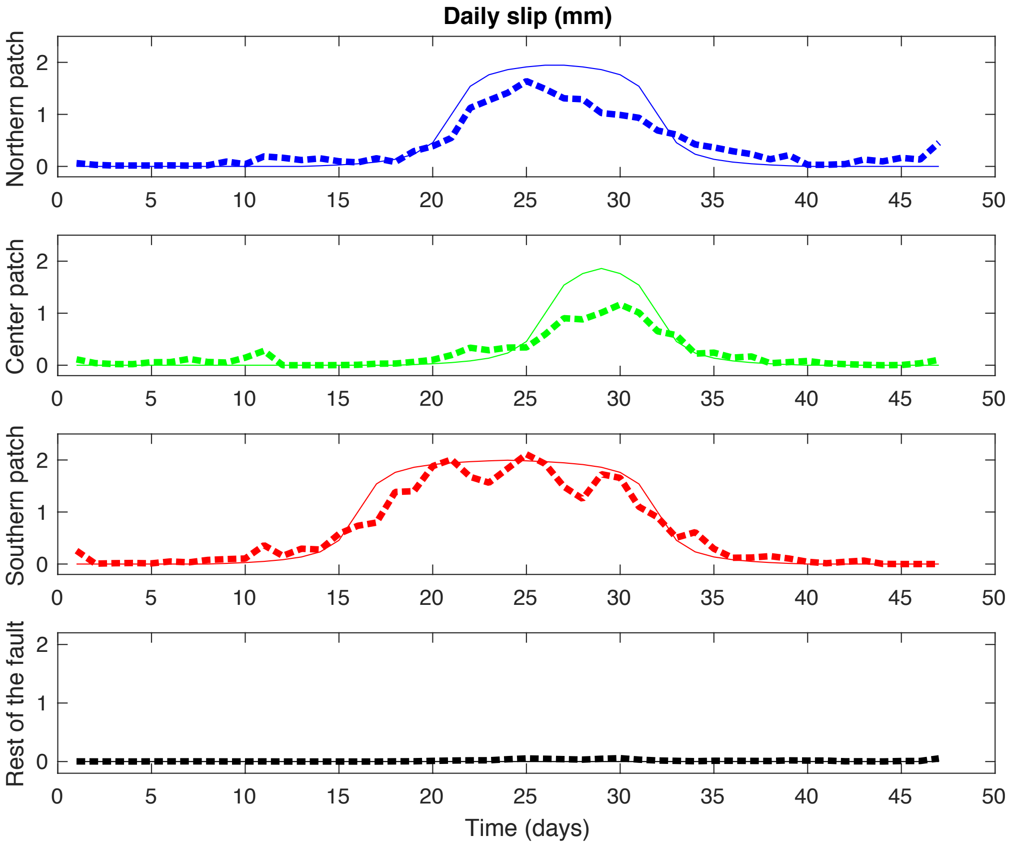

Supplementary Figure 21: Target (plain lines) and recovered (dashed lines) daily slip time series for the 3 patches in the resolution test and the rest of the fault.

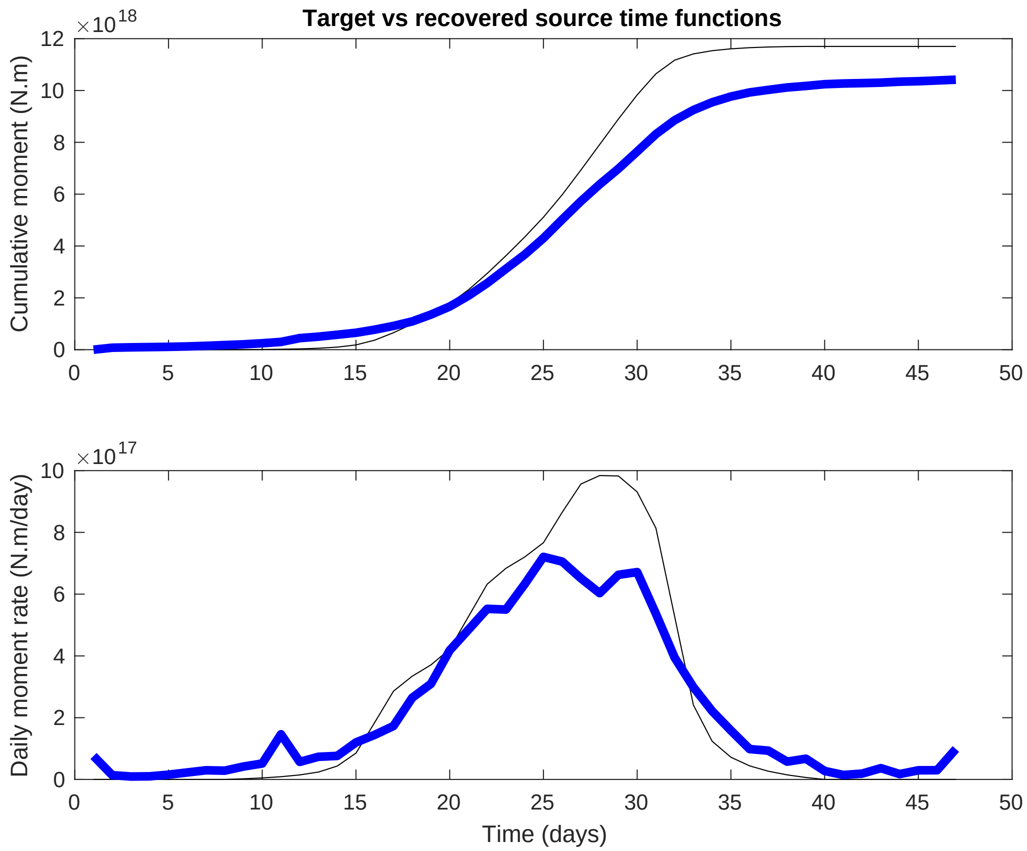

Supplementary Figure 22: Target (thin black lines) and recovered (blue thick lines) cumulative (up) and daily (bottom) source time functions in the resolution test.

References

39. T. A. Herring, *et al.*, *Reviews of Geophysics* **54**, 759 (2016).

40. S. D. Williams, *et al.*, *Journal of Geophysical Research: Solid Earth* **109** (2004).
