# Supplementary material for: Slip bursts during coalescence of slow slip events in Cascadia
Source: Nat Commun. 2020 May 1;11:2159. doi: 10.1038/s41467-020-15494-4 (PMC7195424; doi:10.1038/s41467-020-15494-4)
Supplement: Supplementary file 3 — Description of Additional Supplementary Files [file 41467_2020_15494_MOESM3_ESM.pdf]

Description of Additional Supplementary Files

File name: Supplementary Movie 1
Description: Daily slip velocity during the entire slow slip sequence. Dates are given at the top of the map. Color maps represent slip velocities, blue dots indicate tremors.
